# Seasonal effects of farmer‐managed livestock grazing exclusions on bird communities in Burkina Faso

**DOI:** 10.1002/eap.70160

**Published:** 2025-12-08

**Authors:** Ian Quintas, Gabriel Marcacci, Ambroise N. Zongo, Pius Korner, Alexandra Kuttnig, Reto Spaar, Bakary Diakité, Franziska Kaguembèga‐Müller, Alain Jacot

**Affiliations:** ^1^ Swiss Ornithological Institute Sempach Switzerland; ^2^ Department of Ecology and Evolution University of Lausanne Lausanne Switzerland; ^3^ Association tiipaalga Ouagadougou Burkina Faso; ^4^ Department of Environmental Sciences University of Basel Basel Switzerland; ^5^ newTree Bern Switzerland

**Keywords:** cattle farming, ecological refuge, habitat restoration, land degradation, passive acoustic monitoring, Sahel, seasonality, sustainable use of natural resources

## Abstract

Anthropogenic activities such as livestock grazing and deforestation are primary causes of land degradation in drylands such as the Sahel Zone of Africa, threatening the livelihoods of rural communities and biodiversity. To restore degraded habitats, measures such as farmer‐managed livestock grazing exclusion, where the native vegetation is protected and can naturally regenerate, have been implemented. Yet the benefits of such measures for biodiversity remain poorly understood, especially in regions that experience strong seasonality. Here, we used passive acoustic monitoring to study how livestock grazing exclusion affects the avifauna at the community and species level across the dry and wet seasons. Focusing on an NGO‐driven initiative that has implemented a large network of small‐scale farmer‐managed grazing exclusions in Burkina Faso, we show that species richness and occurrence probability of most bird species were higher in grazing exclusions compared to control sites. These positive effects were more pronounced during the dry season, suggesting an ecological refuge effect when resources are limited. Despite overall positive effects on birds, we found species‐ and guild‐specific responses to grazing exclusion with species positively or negatively affected. While grazing exclusions typically had negative effects on open‐habitat specialists, frugivores, insectivores, species associated with woodlands, and Afro‐Palearctic migratory species were winners. Grazing exclusions, even at small scale, show a great potential to combat desertification, reverse land degradation and halt biodiversity loss thereby being in line with the UN Decade on Ecosystem Restoration. Yet, we emphasize the need for further studies including a socioeconomic perspective to ensure durable benefits for rural communities.

## Résumé

Les activités anthropiques telles que l'élevage et la déforestation sont les principales causes de la dégradation des terres dans les zones arides telles que le Sahel en Afrique, menaçant les moyens de subsistance des communautés rurales et la biodiversité. Pour restaurer les terres dégradées, des mesures ont été mises en œuvre, telles que l'exclusion du bétail de certaines zones par les mises en défens, permettant ainsi à la végétation indigène de se régénérer naturellement. Cependant, les avantages de ces mesures pour la biodiversité restent mal compris, en particulier dans les régions qui connaissent une forte saisonnalité. Ici, nous avons utilisé la méthode dite de monitoring acoustique passif pour étudier comment les mises en défens affectent l'avifaune au niveau de la communauté et des espèces au cours des saisons sèches et humides. En nous concentrant sur une initiative menée par une ONG qui a mis en place un vaste réseau de mises en défens de petites tailles gérées par les agriculteurs au Burkina Faso, nous montrons que la richesse spécifique et la probabilité d'occurrence de la plupart des espèces d'oiseaux sont plus élevées dans les mises en défens par rapport aux sites témoins. Ces effets positifs étaient plus prononcés pendant la saison sèche, suggérant un effet de refuge écologique lorsque les ressources sont limitées. Malgré les effets positifs globaux sur les oiseaux, nous avons constaté que les effets des mises en défens étaient différents selon les espèces et les guildes, avec des espèces affectées positivement ou négativement. Alors que les espèces spécialistes des habitats ouverts étaient typiquement affectées négativement par les mises en défens, les frugivores, les insectivores, les espèces associées aux milieux boisés et les espèces migratrices afro‐paléarctiques étaient gagnantes. Les mises en défens, même à petite échelle, présentent un grand potentiel pour lutter contre la désertification et la dégradation des terres et stopper la perte de biodiversité, conformément à l'appel de la Décennie des Nations Unies pour la restauration des écosystèmes. Cependant, nous insistons sur la nécessité de mener des études plus approfondies, y compris avec une perspective socio‐économique, afin de garantir des avantages durables pour les communautés rurales.

## INTRODUCTION

Human activities such as cattle farming and deforestation are known to pose a major threat to biodiversity and alter ecosystem functions and services (Mach et al., 2015; Pecl et al., 2017; Sponsel, 2013). This has resulted in ongoing land degradation, leading to a global impoverishment of ecosystems and their associated services (AbdelRahman, 2023). Such processes are exacerbated in arid rangelands by a combination of harsh abiotic conditions (especially during the dry season), where even small disturbances can have devastating effects (Anderson, 2005). In the Sahel region, an arid savannah and transition zone stretching from the Sahara Desert in the north to the more humid Sudanian savannahs in the south, land degradation has been identified as one of the major environmental challenges (UNEP, 2012). It is caused by a combination of several factors such as climate change, pressure from population growth, the resulting agricultural expansion, and an increase in cattle farming and unsustainable use of natural resources (e.g., deforestation for firewood/fuelwood collection; Doso Jnr, 2014; Maestre et al., 2022). In particular, the increase in livestock numbers leads to overgrazing, trampling, erosion, and alterations of nutrient cycles, thereby reducing ecosystem functionality and the provision of essential ecosystem services (Barzan et al., 2021; Díaz et al., 2007; Herrero‐Jáuregui & Oesterheld, 2018; Zwarts et al., 2018). These factors impact local farming populations and their livelihoods directly as the soils become increasingly less productive, compromising their food security and their very own subsistence (Barbier & Hochard, 2018; Bielders et al., 2002), thus increasing their vulnerability to ongoing global changes.

Mitigation measures against land degradation that focus on sustainable land management to restore habitats which are in line with the framework of the UN Decade on Ecosystem Restoration are urgently needed (Di Sacco et al., 2021; UNEP, 2021). In the Sahel region, they partly fall within the large‐scale framework of the Great Green Wall Initiative (GGWI) whose goals are to restore 100 million hectares of degraded land and to create a green belt from Senegal to Djibouti (Goffner et al., 2019; http://www.grandemurailleverte.org/). One promising method to restore degraded lands is excluding livestock grazing to enable natural regeneration of the vegetation in combination with sustainable use of natural resources (Barzan et al., 2021; Diop et al., 2024; Forrester et al., 2017; Marcacci, Kaboré, et al., 2025). This strategy has proven its effectiveness in different geographic regions such as North American rangelands (Fühlendorf & Engel, Fühlendorf & Engle, 2001), South American grasslands (Fedrigo et al., 2018) and forests (Etchebarne & Brazeiro, 2016), or Ethiopian woodlands (Yaebiyo et al., 2024). Yet these studies did not account for temporal variations across different seasons and their effects on biodiversity recovery. Given the high seasonality of the Sahel region, the effectiveness of livestock grazing exclusion across wet and dry seasons remains unstudied (Fedrigo et al., 2018; Nabinger & de Faccio Carvalho, 2009). Moreover, the success of these restoration initiatives is often measured solely in terms of vegetation recovery, neglecting other dimensions of the ecosystem and its biodiversity despite its critical importance for ecosystem functioning (Maestre et al., 2022; Majer, 2009; Ortega‐Álvarez & Lindig‐Cisneros, 2012; Young, 2000). Faunal diversity needs to be evaluated as animals have major functions in ecosystems, such as pollination, seed dispersal, pest control, and ecosystem engineering (Ortega‐Álvarez & Lindig‐Cisneros, 2012; Sekercioglu, 2006; Whelan et al., 2008). This is particularly the case for birds which have been increasingly used as indicator species in ecological restoration research—although not yet in the Sahel—as they are considered to provide multiple services and to be mobile links of primary importance (Lundberg & Moberg, 2003; Whelan et al., 2008). Furthermore, birds, as a highly mobile taxa, are regarded as early responders to habitat restoration, can be surveyed at a low cost over large areas (Chowfin & Leslie, 2021; Sekercioglu, 2006) and, given their specific ecological requirements, can inform about distinct aspects of ecosystem health (Ortega‐Álvarez & Lindig‐Cisneros, 2012). Understanding the importance of restored habitats for bird communities across different seasons is therefore crucial, especially when resources are most limited. Furthermore, not all species can benefit from grazing exclusions, with varying effects depending on their ecological requirements (e.g., habitat and diet preferences) and life histories (e.g., migrant vs. resident species).

In this context, the Swiss‐based organization *newTree* (www.newtree.org) and its partner in Burkina Faso *tiipaalga* (www.tiipaalga.org) have been collaborating since 2003 to support rural communities in habitat restoration and sustainable land management efforts. More specifically, *tiipaalga* has implemented a network of farmer‐managed small‐scale grazing exclusions throughout Burkina Faso, where the vegetation is protected from grazing and sustainably managed, allowing recovery of degraded habitat, thus aiming at improving the livelihoods of local rural communities and conserving biodiversity (Marcacci, Kaboré, et al., 2025).

This study aims to show the effects of grazing exclusion on the avifauna in the Sahel region, and how these effects vary between two distinct seasons. More precisely, this study aims to evaluate whether grazing exclusions can benefit bird communities—from the entire community to different guild‐ and species‐specific level—and assess whether these benefits are influenced by the strong seasonality characterizing our study region. Given the current state of knowledge, we hypothesize that bird communities are more diverse with more species having a higher occurrence probability in grazing exclusions and that these responses are exacerbated during the dry season when resources are scarcer across the landscape. We further expect guild‐specific responses to grazing exclusions, for example, decreased occurrence probability for open‐habitat species. The evaluation of these hypotheses is crucial as it can demonstrate the potential of grazing exclusions as a promising restoration measure that enhances biodiversity while benefiting rural communities, thus guiding future restoration efforts that can be initiated elsewhere in the Sahel zone.

## MATERIALS AND METHODS

### Study area

**FIGURE 1:**
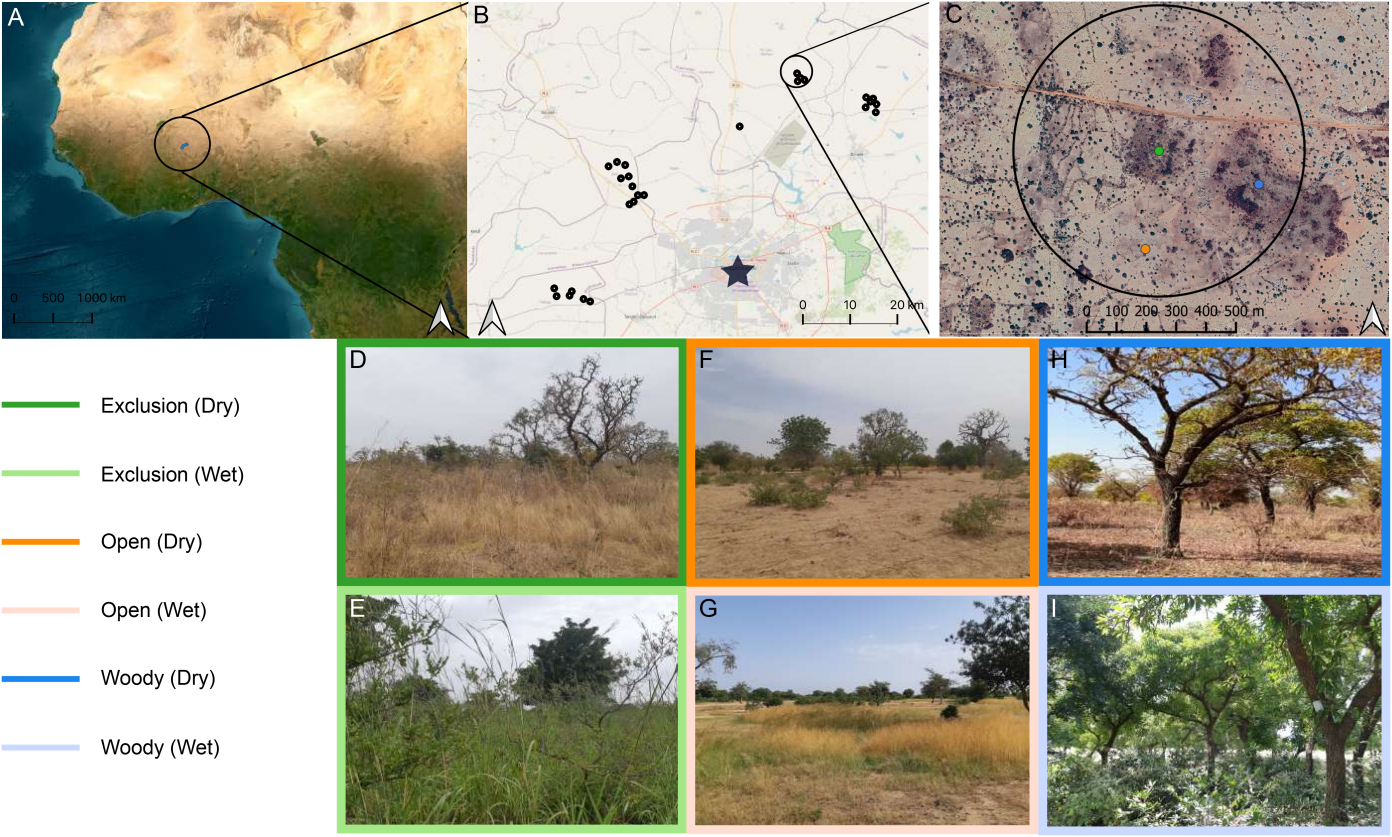
(A) Location of the study area. (B) Location of the 27 landscape units around the capital city Ouagadougou (marked with a black star). (C) Example of a landscape unit (black circle) comprising a grazing exclusion (exclosure, green dot) and the two control sites (blue dot for woody and orange dot for open controls, respectively). The dots indicate where the autonomous recording units (ARUs) were placed. Pictures of typical vegetation found in the grazing exclusions (D, E), open (F, G) and woody controls (H, I) in the dry and wet seasons. Satellite images from GoogleMaps. Photo credits: Gabriel Marcacci.

The study was carried out in three administrative regions of Burkina Faso, namely, Centre, Centre Ouest, and Plateau Central. These regions are situated within the Sudano‐Sahelian ecological zone, which is a transitional zone between the arid Sahel to the north and the more humid Sudanian savannah to the south (Figure 1A). This climatic gradient influences the vegetation cover of the landscape surrounding our study sites with more developed vegetation in the south. In the study area, the climate is characterized by two distinct seasons: a rainy season spanning from July to October and a prolonged dry season from November to June. The vegetation undergoes substantial transformations as the dry season progresses from November, when the vegetation is all green after the rains, through June. As in several other countries of the Sahelian region, Burkina Faso is undergoing a rapid increase in population growth (2022: 22.9 million people vs. 40.1 million (+75%) predicted in 2050 by https://www.prb.org/international/geography/burkina-faso/), which will likely result in an increase in livestock farming and deforestation, thus increasing the current stress on ecosystems. It is estimated that currently, the majority of the Burkina Faso population (68%) relies on small‐scale agriculture (from The World Bank https://data.worldbank.org/indicator/SP.RUR.TOTL.ZS?locations=BF) and 80% of the rural population possesses cattle (Ayantunde et al., 2023).

### Study design

Since 2003, the international organization *newTree* and its local partner, the association *tiipaalga*, have established over 400 grazing exclusions (also called “exclosures” in this study; commonly called “mise en défense” in French) each spanning approximately 3 hectares—in Burkina Faso. All these exclosures are permanently installed and fenced. Their aim is to exclude free‐range livestock grazing to allow the native vegetation to recover naturally. All grazing exclusions are established at the demand of the landowner, who keeps its ownership and manages its grazing exclusion with technical advice and support from *tiipaalga* (see Marcacci, Kaboré, et al., 2025 for more details about the establishment of a grazing exclusion). There are no large wild mammals remaining in the study region; thus, these exclosures cannot have negative impacts on the megafauna. For our research, 27 of these grazing exclusions that were of minimum five years old were selected (see Marcacci, Kaboré, et al., 2025), ensuring that there was a minimum distance of 1 km between any two of them to maintain their independence in terms of the bird communities being sampled (mean distance between grazing exclusions within each locality = 1.6 km ± 1.7 SD; see Marcacci et al., 2021). All selected exclosures are situated near the capital city, Ouagadougou (see map in Figure 1B), for security considerations.

To define a landscape unit for our study, a circular area with a 500‐m radius around the center of each exclosure was designated (see black circles in Figure 1C). Within each landscape unit, two control sites, each of a similar size to the exclosures, were selected. The first control site had a similar vegetation cover as the exclosures (hereafter called “woody”, see blue dots in Figure 1C), while the second control featured a more open habitat (hereafter called “open”, see orange dots in Figure 1C). Initially, remote‐sensing techniques were used, including aerial images and NDVI (Normalized Difference Vegetation Index) data, to screen control sites with varying habitat characteristics, such as tree density and NDVI (Appendix S1: Figures S3 and S4). However, the final selection of the control sites was determined in the field. To ensure their independence in terms of acoustic recordings (so that they do not record the same birds), controls were placed at a mean distance of 295 meters (SD = 92 m) from the grazing exclusions and from each other's centers. This distance (also used in other similar studies: e.g., Karp et al., 2018) allows to compare the habitat use of birds (i.e., which of the three habitat types they use the most) from the same community within each landscape. The two types of control sites enabled us to investigate the impact of vegetation on bird communities (exclosure vs. open) and, additionally, to explore the effects of grazing exclusion, regardless of the overall vegetation cover but due to other intrinsic factors such as differences in vegetation structure and diversity (exclosure vs. woody).

All farmers and landowners granted their approval prior to conducting any research activities on their land.

### Bird survey

Bird surveys were conducted via passive acoustic monitoring (PAM) using Autonomous Recording Units (ARU: AudioMoth Dev 1.2.0; Hill et al., 2019) over two sampling rounds: October to November 2022 and March to April 2023, hereafter called “wet” and “dry”, respectively, corresponding to the “early dry” (or “post rainy”) and “late dry” season (total: 27 sites × 3 habitats × 2 seasons = 162 acoustic surveys; but note that due to technical issues with four recorders, the final dataset contains 158 surveys). The time windows of these two sampling rounds were chosen as they included the overwintering and passing through season for migrant species as well as the breeding season for resident species, while maximizing contrasts in terms of environmental conditions. The first round started right after the end of the rainy season when the vegetation is flourishing and food resources peak and coincides with the arrival and passage of migrant birds and the breeding time of resident birds. The second round was conducted at the end of the dry season, before migrant birds start their journey back to Europe. These two sampling rounds thus allow investigating the effect of temporal variations in the vegetation on the same bird communities, in addition to that of grazing exclusions.

PAM is now becoming a standard approach to survey birds as it is as effective as other standard protocols, such as human transect observations or point counts (Darras et al., 2019). PAM has also proven to be highly effective for monitoring nocturnal, rare, or secretive species which can be challenging to detect (Goyette et al., 2011). It also enables collecting data over large spatial and temporal scales, thus increasing the detection rates of those species and preventing two major biases of conventional bird surveys leading to differences in bird detection probabilities due to sampling time and observers (Bobay et al., 2018; Sugai et al., 2019). Moreover, PAM allows studying hard‐to‐reach regions, because of dense vegetation, elevation, or even security in the case of Burkina Faso (Joel et al., 2024; Serrurier et al., 2024).

For every grazing exclusion and control site, one ARU was placed in the middle of the site, attached to a tree at 1.5 m above ground (see Figure 1C). For each site and season, a total of 2 h was recorded starting 30 min before dawn (start of civil twilight) and ending 90 min after dawn, thus covering the peak activity of most diurnal bird species while also allowing for the detection of nocturnal species. The ARUs were programmed to record birds at a high temporal resolution (1 min recording every 6 min) as this increases detection rates (Metcalf et al., 2022). Overall, we obtained 20 × 1‐min recordings spread over 2 h for every site and season (total: 20 × 1‐min recordings × 162 acoustic surveys = 54 h of acoustic survey). In doing so, the total recording duration of 20 min per site and season is comparable to a standard point count survey, but spanning a larger temporal window, which increases detection probabilities of species with time‐specific vocal activity (Metcalf et al., 2022). To avoid differences in detection ranges between sites due to differences in sound propagation in different habitats, we made sure to verify and standardize detection ranges in grazing exclusions and control sites prior to starting bird surveys, following Darras et al., 2018.

Because automated solutions such as machine learning models (e.g., BirdNET) do not cover all African bird species yet, we identified bird species manually using the software Raven Lite (Yang, 2023; https://ravensoundsoftware.com/) and the online platform ecoSound‐web (Darras et al., 2023; https://ecosound-web.de/ecosound_web/). All bird calls were manually annotated by IQ, AK and AJ, and revised by GM, who has extensive experience in African ornithology. A subset of the annotations, containing unknown species, was revised and validated by external experts (see acknowledgements). Some species were too difficult to identify with certainty only from their vocalizations and were therefore grouped together (i.e., species of genera *Lamprotornis* and *Ploceus*). Out of a total of 19,380 bird detections 159 could not be identified (i.e. less than 1%) and were not considered in the analyses. We extracted lists of all species detected in every 1‐min recording from which we derived overall species richness (total number of species detected) and species‐specific occurrence probability for each study site and season. Species were further grouped into guilds according to their preferred diet and habitat, and migration strategy using the online library AVONET (Tobias et al., 2022; and see Appendix S1: Table S1).

### Vegetation survey

The vegetation of each grazing exclusion and control site was surveyed in October 2022 by a field technician of *tiipaalga* (ANZ), who is an expert in tree inventories. In each site, he surveyed all tree species and their number of stems within a radius of 30 meters around the ARU, for four vegetation strata (below 1 m, between 1 and 2 m, between 2 and 5 m and above 5 m). The Shannon diversity index was calculated across all strata in order to quantify the vertical vegetation heterogeneity and complexity following Vollstädt et al. (2018). The coverage and mean height of herbaceous vegetation were estimated within a 10‐m radius around the ARU. The tree inventory was only conducted once, whereas the herbaceous vegetation variables were measured for each season to account for temporal changes.

### Landscape‐scale variables

Remote sensing was used to calculate vegetation cover inside every exclosure and control site (within a 50‐m radius) but also for each 500‐m radius landscape unit, informing about the surrounding landscape vegetation. To do so, the NDVI was chosen as it is a good proxy for the greenness of the vegetation and above‐ground primary production, especially in rangeland ecosystems where the vegetation is limited (Maestre et al., 2022). The NDVI was calculated from five satellite images (Sentinel‐2 with a 10‐m resolution) for each season and the mean, median, and maximum values were computed. Because NDVI values were lowered due to clouds and dust, we only considered max NDVI for the analyses. For each landscape unit, the difference of max NDVI between wet and dry seasons was calculated to test the effect of NDVI regardless of the season.

In addition, we manually counted the number of farming houses within a landscape unit using satellite images. Because almost all farming households own livestock, we used this number as a proxy for grazing pressure.

### Statistical analyses

#### Effect of grazing exclusion on bird richness

First, we investigated the effect of grazing exclusions on the overall bird community. To do so, we regressed species richness against the habitat type (exclosure, woody, open) in interaction with season (wet and dry), and against other environmental variables. Prior to fitting the model, we performed correlation tests to check for collinearity between environmental variables with a threshold of |*r*_s_| > 0.5. When two variables were strongly correlated, we kept the most biologically meaningful of them. We retained for the model the difference of max NDVI in a landscape unit (NDVI within a radius of 500 m) between the two seasons, the number of trees (within a 30‐meters radius around the ARU), vertical vegetation heterogeneity (Shannon index), herbaceous vegetation cover (%) (within a 10‐meters radius around the ARU), and grazing pressure (number of farming households within a radius of 500 meters). We added the age of the grazing exclusion (number of years since its establishment) as a covariate in the model. All numeric variables were standardized (mean = 0, SD = 1) to improve convergence of the models. We fitted a Bayesian generalized linear mixed‐effects model assuming a Poisson data distribution and random intercepts per landscape unit to account for the spatial clustering of our study design. We additionally checked that there was no spatial autocorrelation of the residuals (Moran's I test: *p*‐value = 0.54).

#### Species‐specific responses to grazing exclusion

A common drawback when using PAM is that it is difficult to detect individual calling animals and assess species abundances (Gibb et al., 2019). When dealing with presence/absence data, occupancy models are often used due to the link between species occupancy and abundance (Gaston et al., 2000). Such models require multiple surveys of the same site within a short time window (Kéry & Royle, 2016). But due to security reasons and to keep the ARUs from being stolen, we could only perform one survey per site and season. Instead, we conducted a second analysis using the number of detections (in our study, the number of detections corresponds to the number of 1‐min recordings in which a species was detected) for each species in each site and season to estimate the occurrence probability of each species. Although this model does not account for imperfect detection like occupancy models, using PAM reduces some of the biases commonly associated with bird survey data (i.e., differences in detection probabilities between different observers or survey times). Because the vocal activity is species‐specific (e.g., not all species sing at the same time), we conducted the analysis using a different time interval (within the 20 × 1‐min recordings spread across the 2‐h morning period) for every species. This time interval was defined as the interval from the first to the last 1‐min recording having at least a minimal probability for detection (we used a threshold of >0.001). To calculate minimal detection probabilities, we fitted a logistic regression per species, with detection/non‐detection modeled against the 20 × 1‐min recordings, used as a factor with 20 levels. Only species with at least 30 detections in total were considered for this analysis, that is, 55 out of the total of 87 recorded species.

After this selection of the relevant time interval per species, the occurrence probability was modeled (one model including all species) using a Bayesian binomial generalized linear mixed model with the number of detections and non‐detections within the species‐specific time intervals as the response variable. Habitat and season were used as fixed effects with an interaction between them. This interaction was also used to model random slope effects per species to allow for species‐specific responses. Further, random intercepts were estimated per landscape unit to account for the spatial clustering of the study design. We also included an observation‐level random intercept to account for overdispersion and zero‐inflation of the data (Korner‐Nievergelt et al., 2015). The estimated occurrence probabilities for each species were compared between grazing exclusion and the two control sites (exclosure vs. open/exclosure vs. woody) and for both seasons. To do so, we used the predicted difference of occurrence probability between grazing exclusions and control sites for both seasons (including error propagation to calculate a 95% credible interval CrI for the difference, using the function *posterior_epred* from the R‐package *rstantools* (Gabry et al., 2022)). If the difference was positive and 0 not in the 95% CrI, the species was considered positively affected by grazing exclusion or negatively in the opposite case. When the CrI overlapped 0, the species was classified as “unclear” (effect of exclosure).

#### Guild‐specific responses to grazing exclusion

Finally, we modeled the effects of grazing exclusion on different species groups or guilds. Three different nonexclusive guilds were investigated for all species, namely their preferred habitat (open/woody/intermediate), preferred diet (frugivore/granivore/invertivore/other) and migration strategy (resident/migrant). We selected these specific traits as (1) understanding the preferred habitat helps evaluate the restoration's effectiveness in providing suitable conditions for diverse ecological niches (Alsila et al., 2021), (2) investigating trophic niches allows us to assess the availability of food resources and potential provision of (dis)services (Sekercioglu, 2006), and (3) considering migration strategies is crucial due to the critical importance of the Sahel region for many migratory birds (Marcacci et al., 2023).

The multispecies model described above was adapted to analyze guild‐specific responses by adding an ecological trait as a fixed effect, including the three‐way interaction habitat × season × trait, with random intercepts estimated per landscape unit to account for the spatial clustering of the study design and an observation‐level random intercept to account for overdispersion and zero‐inflation of the data. One model for each ecological trait (habitat preference, diet, and migration) was fitted, using the same structure for each.

All statistical analyses were performed in R version 4.2.1 (R Core Team, 2023). All Bayesian models were fitted with the function *stan_glmer* from the *rstanarm* R‐package (Goodrich et al., 2023) with 6000 iterations, thereof 2000 burn‐ins, four chains, and non‐informative priors. Model diagnostics and assumptions (Rhat values, trace plots, posterior predictive distribution plots) were checked, using the functions *summary*, *plotfun*, and *pp_check* from the *rstanarm* R‐package.

## RESULTS

Across all sites and seasons we recorded 19,380 detections (= detection of a species within a 1‐min recording) of 87 species (see Appendix S1: Table S1). We estimated sampling completeness using the Chao 1 estimator, which indicated that we sampled 89.7% and 91.7% (in the dry season and the wet season respectively) of the bird species present in the study sites. Seventy species are residents of West Africa and 17 are migrants (Borrow, 2020). The 10 most recorded species were common bulbul *Pycnonotus barbatus* (2148 detections = number of 1‐min recordings the species was present), vinaceous dove *Streptopelia vinacea* (2142), laughing dove *Spilopelia senegalensis* (1395), yellow‐crowned gonolek *Laniarius barbarus* (1324), northern gray‐headed sparrow *Passer griseus* (1125), black‐billed wood‐dove *Turtur abyssinicus* (799), yellow‐fronted tinkerbird *Pogoniulus chrysoconus* (790), northern red‐billed hornbill *Tockus erythrorhynchus* (702), red‐cheeked cordonbleu *Uraeginthus bengalus* (659), and tawny‐flanked prinia *Prinia subflava* (608). These 10 species accounted for more than half of all detections recorded (60%). Sites had an average of 122.6 ± 74.0 (SD) detections and a mean of 22.8 ± 4.2 species. The busiest 1‐min recording was the 9th after the beginning of the recordings (i.e. 54 min after dawn) with a total of 1330 detections across all sites. Regarding the total number of recorded detections, 3051 and 4798 detections were registered in the grazing exclusions for the dry and wet season, respectively, 2584 and 3408 detections for the woody control sites, and 2254 and 3285 detections for the open sites.

### Seasonal effects of grazing exclusion on bird richness

**FIGURE 2:**
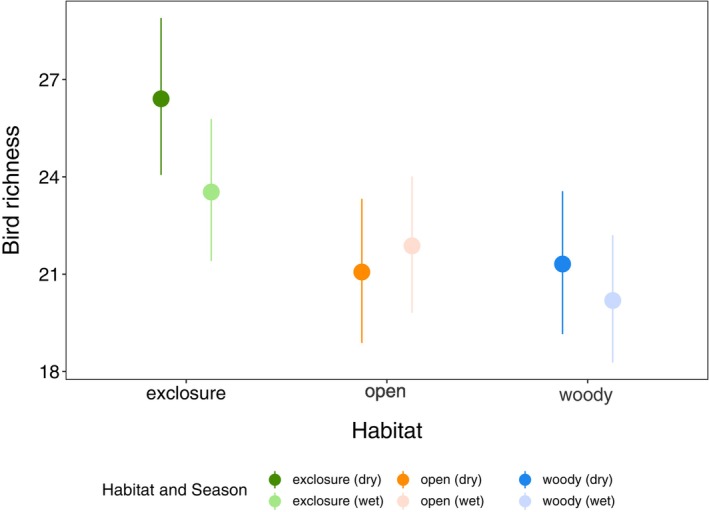
Estimated bird richness in grazing exclusions and the two controls for the dry and wet seasons. Points display the estimated means and the error bars the 95% credible intervals based on a Bayesian Poisson generalized linear mixed‐effects model.

We found a positive effect of grazing exclusion on bird richness that varied between seasons (Figure 2), with a stronger positive effect of grazing exclusions compared to open controls during the dry season than during the wet season. More specifically, compared to woody controls, grazing exclusions had on average 18.9% (95% CrI: [6.4%, 32.9%]) more species of birds in the dry season and 19.3% [6.9%, 33.9%] in the wet season. The positive effect of grazing exclusions compared to open controls was more pronounced in the dry season, where species richness was 29.3% [15.9%, 44.5%] higher, than in the wet season where it was only 13.01% higher [0.9%, 13.1%].

The other covariates included in the model (number of trees, vegetation complexity, number of houses, NDVI, herbaceous coverage, age of the grazing exclusion) had no or little effect on bird species richness (see Appendix S1: Table S2 and Figure S1).

### Species‐specific responses to grazing exclusion

**FIGURE 3:**
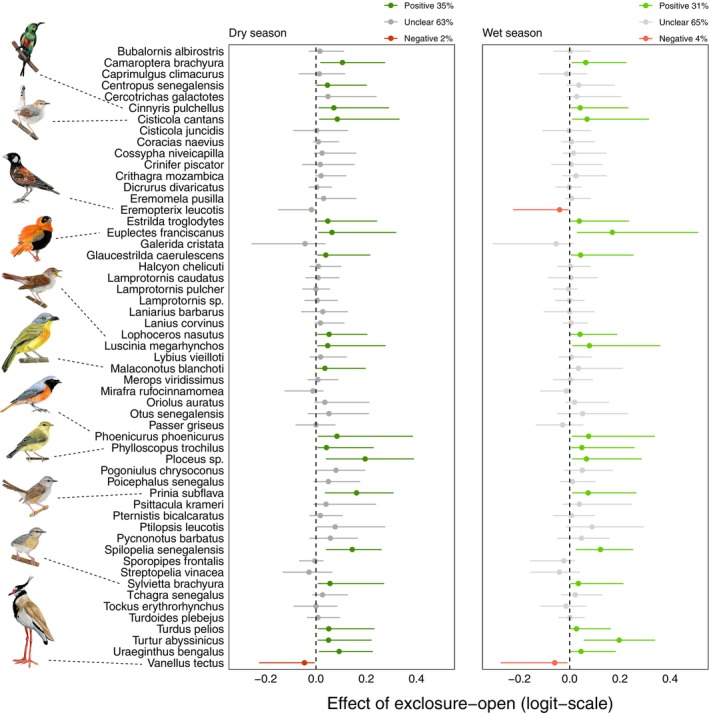
Tree plots showing the estimated effects (with 95% credible intervals) of grazing exclusion compared to open controls for 55 species in the dry and wet seasons. The color is determined by whether the estimated effect is likely different between grazing exclusions and open controls (95% CrI not overlapping 0), with positive effects of grazing exclusions shown in green and negative effects in red. For unclear effects (95% CI overlapping 0), the line is colored in gray. Above each plot, the percentage of species for which grazing exclusion had a positive, negative of unclear effect is indicated. Bird drawings by Alexandra Kuttnig illustrate species mentioned in the text.

**FIGURE 4:**
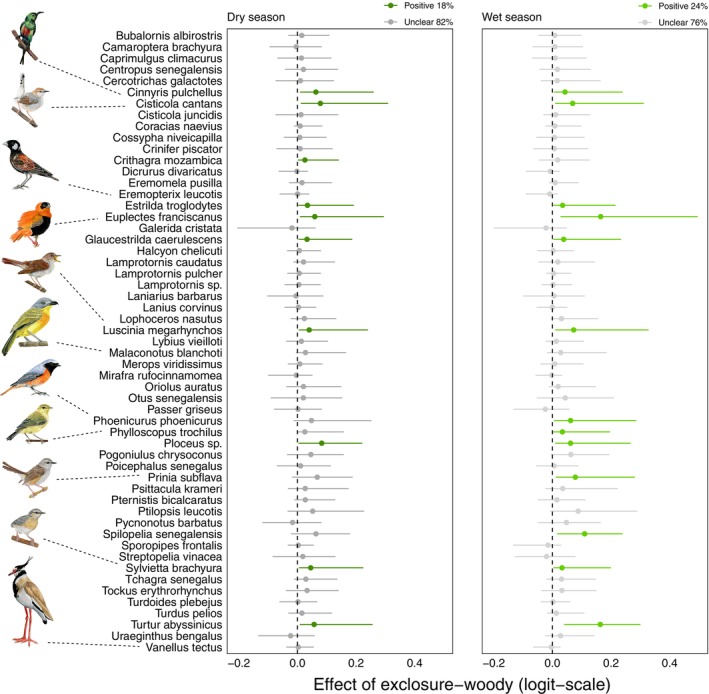
Tree plots showing the estimated effects (with 95% credible intervals) of grazing exclusion compared to woody controls for 55 species in the dry and wet seasons. The color is determined by whether the estimated effect is likely different between grazing exclusions and woody controls (95% CrI not overlapping 0), with positive effects of grazing exclusions shown in green and negative effects in red. For unclear effects (95% CI overlapping 0), the line is colored in gray. Above each plot, the percentage of species for which grazing exclusion had a positive, negative of unclear effect is indicated. Bird drawings by Alexandra Kuttnig illustrate species mentioned in the text.

We found that compared to open controls, there were more species positively affected by grazing exclusions (19 species in the dry season, 17 wet) and only a few negatively affected (1 dry, 2 wet), while the rest of the species were classified as “unclear” (35 dry, 36 wet) (Figure 3). The same comparison was made for woody controls, and it was found that more species were positively affected (10 dry, 13 wet) while none were negatively affected. The rest of the species (45 dry, 39 wet) were classified as “unclear” (Figure 4).

### Guild‐specific responses to grazing exclusion

**FIGURE 5:**
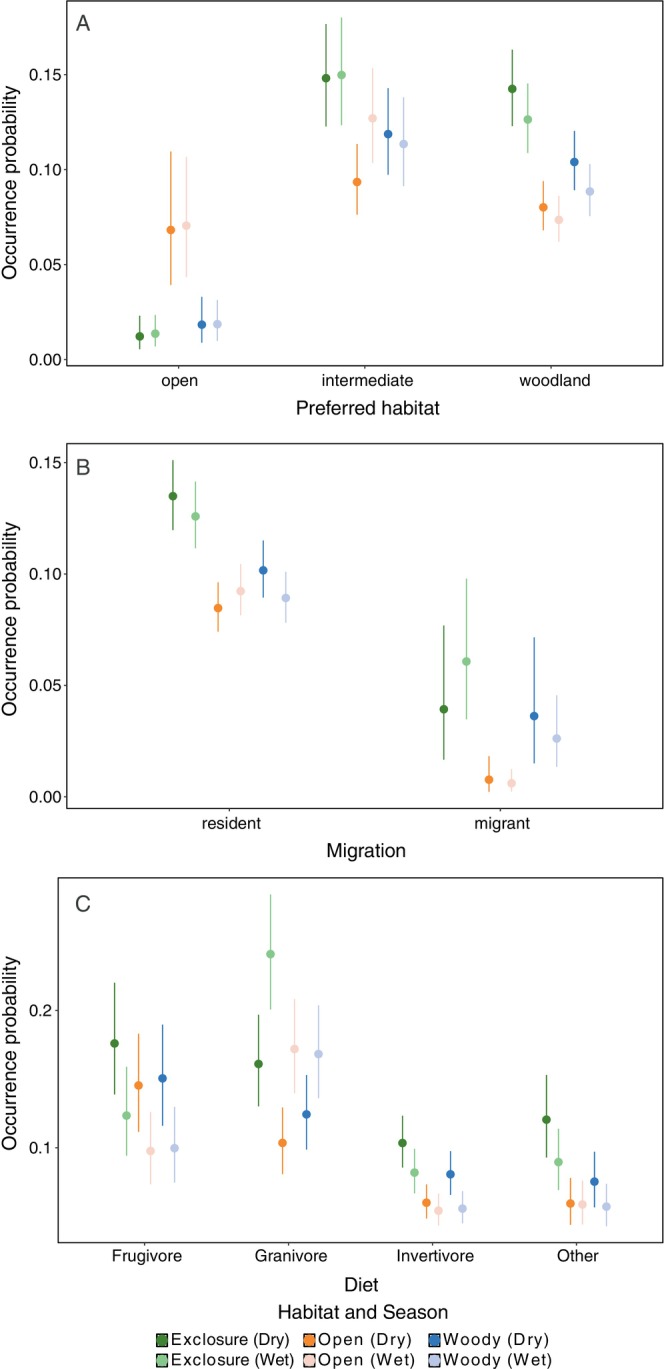
Occurrence probability of species from different guilds in grazing exclusions and control sites in each season. Points display the estimated means and the error bars the 95% credible intervals from Bayesian binomial generalized linear mixed‐effects models. Plots A, B, and C show the estimates for guilds classified according to habitat preferences, migration strategy, and preferred diet, respectively.

For many guilds, the occurrence probability was highest in grazing exclusions during the dry season, reinforcing results from the previous species‐specific analyses (Figure 5). However, there are notable exceptions to this trend. Open‐habitat species had a higher occurrence probability in open control sites, regardless of the season (Figure 5A). Migratory birds had a higher occurrence probability in grazing exclusions during the wet season, but in the dry season, occurrence probability was similar compared to woody controls (see Figure 5B). Overall, migratory birds preferred grazing exclusions and woody controls compared to open controls. Granivorous species were more present during the wet season regardless of the habitat while it was the opposite for frugivores, invertivores, and “others” (see Figure 5C).

## DISCUSSION

In this study, we investigated the effects of small‐scale farmer‐managed livestock grazing exclusion on bird communities in an  understudied region of the world, the African Sahel, which is characterized by two contrasting seasons. Our results demonstrate that grazing exclusions proved successful in promoting both species richness and the occurrence probability of most bird species compared to control sites. These positive effects were exacerbated during the dry season, suggesting that grazing exclusions act as ecological refuges when resources are limited. These findings emphasize how livestock‐free areas, allowing natural regeneration, play a crucial role in supporting biodiversity, particularly during the dry season.

### Grazing exclusion benefits bird communities

At the community level, we found that in both seasons, there was a higher bird species richness in grazing exclusions compared to control sites. This result is in line with other studies that showed that habitat restoration through livestock grazing exclusion can promote bird diversity (Barzan et al., 2021; Dettenmaier et al., 2017; Diop et al., 2024; Ortega‐Álvarez & Lindig‐Cisneros, 2012; Schieltz & Rubenstein, 2016). Tree cover and vegetation complexity are known to have a positive effect on bird diversity (Marcacci et al., 2020; Marcacci et al., 2022; Moudrý et al., 2021). Yet, although grazing exclusions have a more diverse, dense, and complex vegetation structure than control sites (see Appendix S1: Figuress S2–S4, at least in comparison to open controls), we did not find strong effects of the habitat characteristics we measured on bird richness (see Appendix S1: Figure S1). This suggests that the environmental variables such as tree density, herbaceous cover, and NDVI may affect species richness in more complex ways than we explored, for example, via combinations of these variables and other factors associated with grazing exclusion that we did not measure. For example, critical resources such as a higher number of fruiting trees might be more available in exclosures than in the surrounding habitats. One way to better understand how grazing exclusion benefits bird communities is to identify which species of trees are preferred by birds across the seasons (e.g., see Zwarts et al., 2023a). Having this knowledge would also allow promoting these key tree species outside of grazing exclusions. Another possible benefit of grazing exclusions is that they offer a habitat with reduced human disturbances and associated threats, potentially benefiting sensitive species (e.g., hunted species such as partridges and francolins). Finally, it is worth noting that the age of the grazing exclusion had no effect on bird richness, suggesting that even 5‐year‐old grazing exclusions can already benefit avian communities. Note that our results demonstrate a positive effect of grazing exclusions on bird communities, which used more exclosures (e.g., for foraging) than the grazed surrounding landscapes; while we cannot confirm this with our data, we do believe that many species also profit from grazing exclusions for breeding.

### Grazing exclusions act as an ecological refuge during the dry season

The positive effect of grazing exclusions is exacerbated during the dry season, strongly suggesting that they act as ecological refuges throughout the degraded landscape, especially when resource availability is limited (Zwarts et al., 2023b) (Figure 2). Birds in the direct surrounding landscapes of grazing exclusions may shift habitats and concentrate in restored areas during the dry season. It seems likely that the refuge effect is due to a reduced vegetation cover during the dry season outside of exclosures, caused by the combination of unsustainable livestock grazing and a lack of precipitation (Doso Jnr, 2014; Herrmann et al., 2005; Ortega‐Álvarez & Lindig‐Cisneros, 2012). Restored habitats through grazing exclusions, even at a small scale as highlighted in this study, appear to provide sufficient resources for multiple bird species to survive the dry season in these landscapes. In contrast, during the wet season, herbaceous vegetation can persist in degraded and overgrazed habitats, leading to more suitable habitats for birds and thereby also reducing the magnitude of the positive effects of restored habitats.

When investigating the ecological refuge effect at the species‐specific level, we see that the results obtained at the community level are reinforced. Indeed, many species were positively affected by grazing exclusions (i.e., higher occurrence probability in grazing exclusions than in control sites) during the dry season showcasing the positive effect of grazing exclusions (Figures 3 and 4). Furthermore, several species changed their status from *negative* or *unclear* to *positive* between the wet and dry seasons, suggesting local movements to track resource availability leading to a shift in preferred habitats. For example, the gray‐headed bushshrike (*Malaconotus blanchoti*), a species more associated with woody areas, was more present in grazing exclusions during the dry season whereas it was equally distributed between habitats during the wet season. This shows how grazing exclusions can have a positive impact on species that would not prefer restored sites under better landscape conditions—during the wet season—but still take advantage of them under harsher conditions—during the dry season. Thus, the ecological refuge effect may also be important for species that are not necessarily adapted to the ecosystem provided by grazing exclusions.

Nevertheless, we have to keep in mind that we monitored birds' vocalizations and that it is not possible to disentangle vocal inactivity from true absences. It is therefore possible that part of the seasonal effect encountered in this study is explained by the difference in vocal activity which cannot be accounted for when using PAM. However, it seems unlikely that it would affect our results regarding the impact of grazing exclusions as this bias (varying detection probability between seasons) would likely be similar for each habitat. We nonetheless recommend for future studies to (whenever possible) increase the number of surveys per site and sampling round in order to use a modeling approach such as occupancy models that take imperfect detection into account (Kéry & Royle, 2016).

### Winners of grazing exclusions

Many species had a higher occurrence probability in grazing exclusions and could be considered winners (Figures 3 and 4). By contrast, only two species were negatively affected by grazing exclusions: the black‐headed lapwing (*Vanellus tectus*) and the chestnut‐backed sparrow‐lark (*Eremopterix leucotis*), which are adapted to open habitats (Figures 3 and 5A). This pattern is expected, and we need to keep in mind that habitat restoration measures will inevitably have a negative impact on certain bird species that are not adapted to these restored habitats (Alsila et al., 2021). This suggests that other conservation measures for these species need to be applied if necessary.

There were 10 species that systematically benefited from grazing exclusions in all comparisons. From these 10 species, four were insectivores (singing cisticola *Cisticola cantans*, common nightingale *Luscinia megarhynchos*, tawny‐flanked prinia *Prinia subflava* and northern crombec *Sylvietta brachyura*), suggesting grazing exclusions provide more food resources across seasons. Out of the 10 species, only one was an open‐habitat specialist, the northern red bishop (*Euplectes franciscanus*). It was almost exclusively found in grazing exclusions as this bird species is closely dependent on tall grasses and dense herbaceous vegetation, explaining why grazing exclusions have such a beneficial impact on it and why it is rarely found in control sites (Borrow, 2020). Another of these species was the beautiful sunbird (*Cinnyris pulchellus*), a nectarivorous species.

Palearctic migrants such as common nightingale (*Luscina megarhynchos*) were also winners of grazing exclusions. Other migrants such as common redstart (*Phoenicurus phoenicurus*) and willow warbler (*Phylloscopus trochilus*) were also more present in grazing exclusions compared to open but not woody controls. This suggests that even in grazed landscapes there may still be enough trees to provide habitat for some Palearctic migrants, as already shown for a related species, the wood warbler (*Phylloscopus sibilatrix*) in Ghana (Mallord et al., 2018), and for the pied flycatcher (*Ficedula hypoleuca*) in Liberia (Bryant et al., 2025). Overall, these results highlight the crucial importance of restoring degraded lands in the nonbreeding grounds of migratory birds, which are particularly at risk (Marcacci et al., 2023; Vickery et al., 2023).

As shown in this section, the response to grazing exclusion is species‐ and guild‐specific. It demonstrates that identifying which individual species or guilds benefit or not from restoration measures seems a promising strategy to define indicator/umbrella species or groups that can be used to evaluate the success or effectiveness of restoration efforts.

### Potential effects on ecosystems functions and services

Simultaneously, acknowledging the positive effect of grazing exclusions on several bird species and knowing that birds act as valuable bioindicators and providers of diverse ecosystem services (Sekercioglu, 2006; Whelan et al., 2008), it is important to explore the reciprocal benefits that local farmers who practice sustainable agriculture may gain from allowing bird communities to thrive within their land. For example, as nectarivorous species play a major role in pollination, which is essential in forest ecosystems, it is likely that the frequent presence of sunbirds is beneficial to the overall health of the ecosystem (Lundberg & Moberg, 2003). Frugivorous bird species also play a crucial role in ecosystems as they are involved in the dissemination of seeds, potentially amplifying the beneficial effects of grazing exclusions on tree recruitment in the surrounding landscapes (Bello et al., 2024; De la Peña‐Domene et al., 2014). Furthermore, insectivorous species were more present in exclosures than in control sites. This could be beneficial for farmers as insectivorous birds are pest‐controlling agents and likely play an important role in increasing yields of harvested plants (Ferreira et al., 2023; Tela et al., 2021). These results are encouraging as these mentioned species groups provide essential ecosystem services and help in the process of habitat restoration (Buba & Jaafar, 2021; Chowfin & Leslie, 2021). Yet, birds can also provide disservices, such as granivorous species raiding cereal crops, sometimes even posing food security problems for subsistence farmers (Hiron et al., 2014). Further studies must evaluate the role of birds in providing (dis)services to farming households owning a grazing exclusion before drawing general conclusions. To change people's attitudes toward birds, it is essential to understand their perceptions, and showcasing the overall benefits provided by birds is a promising way to achieve this.

## CONCLUSION

Altogether our results demonstrate that small‐scale farmer‐managed livestock grazing exclusions and natural regeneration of the vegetation are highly beneficial for bird communities, showcasing the great potential of this simple restoration measure that can benefit people and biodiversity, in line with the framework of the UN Decade on Ecosystem Restoration. Such measures show a high potential to be upscaled and implemented elsewhere in the Sahel region, for example., under the GGWI. The positive effects of grazing exclusions were stronger during the dry season, suggesting they act as ecological refuges when resources are limited. This highlights that seasonality must be accounted for when evaluating the success of restoration initiatives. Our results also show that PAM is a promising strategy to monitor restoration efforts. With recent and future developments in AI technologies such as automated sound classification, PAM will offer a cost‐effective opportunity to monitor biodiversity recovery over large spatial and temporal scales, making birds efficient indicators of restoration success. What is needed now is to quantify the direct and indirect benefits of grazing exclusions and how they contribute to improving the livelihood of farming households through the provision of multiple ecosystem services, including those provided by birds. Only by adopting a socio‐ecological perspective can we make the Sahel greener for people and biodiversity.

## CONFLICT OF INTEREST STATEMENT

The authors declare no conflicts of interest.

## Supporting information


Appendix S1.

## Data Availability

Data (Marcacci, Quintas, et al., 2025) are available in Zenodo at https://doi.org/10.5281/zenodo.13982871.
