# Supplementary material for: Seasonal effects of farmer‐managed livestock grazing exclusions on bird communities in Burkina Faso
Source: Ecol Appl. 2025 Dec 8;35(8):e70160. doi: 10.1002/eap.70160 (PMC12683984; doi:10.1002/eap.70160)

# Appendix S1

## Seasonal effects of farmer-managed livestock grazing exclusions on bird communities in Burkina Faso

Ian Quintas, Gabriel Marcacci, Ambroise N. Zongo, Pius Korner, Alexandra Kuttnig, Reto

Spaar, Bakary Diakité, Franziska Kaguembèga-Müller, Alain Jacot

***Ecological Applications***

**Table S1.** List of species with their associated traits.

| # | **Species** | **habitat preference** | **migration strategy** | **preferred diet** |
|---|---|---|---|---|
| 1 | *Pycnonotus barbatus* | woodland | resident | Frugivore |
| 2 | *Streptopelia vinacea* | intermediate | resident | Granivore |
| 3 | *Spilopelia senegalensis* | intermediate | resident | Granivore |
| 4 | *Laniarius barbarus* | woodland | resident | Invertivore |
| 5 | *Passer griseus* | intermediate | resident | Granivore |
| 6 | *Turtur abyssinicus* | woodland | resident | Granivore |
| 7 | *Pogoniulus chrysoconus* | woodland | resident | Frugivore |
| 8 | *Tockus erythrorhynchus* | woodland | resident | Omnivore |
| 9 | *Uraeginthus bengalus* | intermediate | resident | Granivore |
| 10 | *Prinia subflava* | woodland | resident | Invertivore |
| 11 | *Camaroptera brachyura* | woodland | resident | Invertivore |
| 12 | *Crithagra mozambica* | intermediate | resident | Omnivore |
| 13 | *Poicephalus senegalus* | woodland | resident | Frugivore |
| 14 | *Tchagra senegalus* | woodland | resident | Invertivore |
| 15 | *Pternistis bicalcaratus* | woodland | resident | Granivore |
| 16 | *Ploceus sp.* | intermediate | resident | Omnivore |
| 17 | *Lamprotornis caudatus* | intermediate | resident | Frugivore |
| 18 | *Caprimulgus climacurus* | intermediate | resident | Invertivore |
| 19 | *Lophoceros nasutus* | woodland | resident | Omnivore |
| 20 | *Bubalornis albirostris* | intermediate | resident | Omnivore |
| 21 | *Centropus senegalensis* | woodland | resident | Vertivore |
| 22 | *Lanius corvinus* | woodland | resident | Invertivore |
| 23 | *Euplectes franciscanus* | open | resident | Granivore |
| 24 | *Lybius vieilloti* | woodland | resident | Frugivore |
| 25 | *Ptilopsis leucotis* | woodland | resident | Vertivore |
| 26 | *Eremomela pusilla* | woodland | resident | Invertivore |
| 27 | *Sporopipes frontalis* | open | resident | Granivore |
| 28 | *Cisticola cantans* | woodland | resident | Invertivore |
| 29 | *Turdus pelios* | woodland | resident | Omnivore |
| 30 | *Merops viridissimus* | intermediate | resident | Invertivore |
| 31 | *Otus senegalensis* | woodland | resident | Invertivore |
| 32 | *Vanellus tectus* | open | resident | Invertivore |
| 33 | *Lamprotornis pulcher* | intermediate | resident | Frugivore |
| 34 | *Cinnyris pulchellus* | woodland | resident | Nectarivore |
| 35 | *Dicrurus divaricatus* | woodland | migrant | Invertivore |
| 36 | *Phylloscopus trochilus* | woodland | migrant | Invertivore |
| 37 | *Sylvietta brachyura* | woodland | resident | Invertivore |
| 38 | *Oriolus auratus* | woodland | resident | Frugivore |
| 39 | *Eremopterix leucotis* | open | resident | Granivore |
| 40 | *Luscinia megarhynchos* | woodland | migrant | Invertivore |

| | | | | |
|---|---|---|---|---|
| 41 | *Cercotrichas galactotes* | woodland | migrant | Invertivore |
| 42 | *Malaconotus blanchoti* | woodland | resident | Invertivore |
| 43 | *Turdoides plebejus* | woodland | resident | Invertivore |
| 44 | *Mirafra rufocinnamomea* | open | resident | Omnivore |
| 45 | *Coracias naevius* | intermediate | resident | Invertivore |
| 46 | *Cossypha niveicapilla* | woodland | resident | Invertivore |
| 47 | *Crinifer piscator* | woodland | resident | Frugivore |
| 48 | *Glaucestrilda caerulescens* | intermediate | resident | Granivore |
| 49 | *Lagonosticta senegala* | intermediate | resident | Granivore |
| 50 | *Estrilda troglodytes* | intermediate | resident | Granivore |
| 51 | *Gymnoris dentata* | intermediate | resident | Granivore |
| 52 | *Phoenicurus phoenicurus* | woodland | migrant | Invertivore |
| 53 | *Galerida modesta* | open | resident | Omnivore |
| 54 | *Lamprotornis sp.* | intermediate | resident | Frugivore |
| 55 | *Psittacula krameri* | woodland | resident | Frugivore |
| 56 | *Oena capensis* | intermediate | resident | Granivore |
| 57 | *Upupa epops* | intermediate | migrant | Invertivore |
| 58 | *Halcyon chelicuti* | woodland | resident | Invertivore |
| 59 | *Cisticola juncidis* | open | resident | Invertivore |
| 60 | *Iduna opaca* | woodland | migrant | Invertivore |
| 61 | *Galerida cristata* | open | resident | Omnivore |
| 62 | *Vidua chalybeata* | intermediate | resident | Granivore |
| 63 | *Coracias abyssinicus* | intermediate | resident | Invertivore |
| 64 | *Ptilopachus petrosus* | woodland | resident | Granivore |
| 65 | *Burhinus senegalensis* | open | resident | Invertivore |
| 66 | *Clamator levaillantii* | woodland | migrant | Invertivore |
| 67 | *Pytilia melba* | woodland | resident | Granivore |
| 68 | *Prionops plumatus* | woodland | resident | Invertivore |
| 69 | *Chrysococcyx caprius* | woodland | resident | Invertivore |
| 70 | *Emberiza goslingi* | intermediate | resident | Granivore |
| 71 | *Chloropicus goertae* | woodland | resident | Invertivore |
| 72 | *Indicator indicator* | woodland | resident | Invertivore |
| 73 | *Nilaus afer* | woodland | resident | Invertivore |
| 74 | *Kaupifalco monogrammicus* | intermediate | resident | Invertivore |
| 75 | *Amadina fasciata* | intermediate | resident | Granivore |
| 76 | *Crithagra leucopygia* | intermediate | resident | Granivore |
| 77 | *Chrysococcyx klaas* | woodland | migrant | Invertivore |
| 78 | *Corvus albus* | intermediate | resident | Omnivore |
| 79 | *Burhinus capensis* | open | resident | Invertivore |
| 80 | *Glaucidium perlatum* | intermediate | resident | Invertivore |
| 81 | *Micronisus gabar* | intermediate | resident | Vertivore |
| 82 | *Bubo cinerascens* | woodland | resident | Vertivore |
| 83 | *Buteo auguralis* | intermediate | resident | Vertivore |
| 84 | *Chalcomitra senegalensis* | woodland | resident | Omnivore |
| 85 | *Merops albicollis* | intermediate | migrant | Invertivore |

| | | | | |
|---|---|---|---|---|
| 86 | *Phylloscopus collybita* | woodland | migrant | Invertivore |
| 87 | *Telophorus sulfureopectus* | woodland | resident | Invertivore |

**Table S2.** Summary (estimates and 95% credible intervals) of the species richness model. Habitat has three levels (“exclosure, “open”, “woody”) and season has two levels (“dry”, “wet”).

| **Predictor** | **mean** | **95% CrI** |
|---|---|---|
| (Intercept) | 3.31 | [3.22, 3.39] |
| habitatopen | -0.28 | [-0.44, -0.13] |
| habitatwoody | -0.18 | [-0.3, -0.06] |
| seasonwet | -0.11 | [-0.23, 0.00] |
| age_exclosure | 0.01 | [-0.03, 0.04] |
| diff.max.NDVI.500 | 0 | [-0.04, 0.04] |
| herb_cov | 0.01 | [-0.04, 0.06] |
| nb_trees | 0 | [-0.04, 0.05] |
| nb_houses | -0.03 | [-0.07, 0.01] |
| veg_heterogeneity | -0.03 | [-0.07, 0.02] |
| habitatopen:seasonwet | 0.13 | [-0.03, 0.29] |
| habitatwoody:seasonwet | -0.01 | [-0.17, 0.15] |
| Sigma[landscape_id :(Intercept)] | 0.00 | [0.00, 0.00] |

**Table S3.** Summary (estimates and 95% credible intervals) of the multispecies model (occurrence probability). Note that only the coefficients for the variables habitat and season as well as their interaction at the community level (mean occurrence across all species) are shown, and not the coefficients for all species. Habitat has three levels (“exclosure, “open”, “woody”) and season has two levels (“dry”, “wet”).

| **Predictor** | **mean** | **95% CrI** |
|---|---|---|
| (Intercept) | -2.77 | [-3.12, -2.44] |
| habitatwoody | 0.18 | [-0.05, 0.42] |
| habitatexclos | 0.58 | [0.34, 0.83] |
| seasonwet | -0.01 | [-0.28, 0.24] |
| habitatwoody:seasonwet | -0.17 | [-0.43, 0.08] |
| habitatexclos:seasonwet | -0.12 | [-0.34, 0.1] |
| Sigma[ol :(Intercept)] | 0.98 | [0.91, 1.06] |
| Sigma[landscape_id :(Intercept)] | 0.02 | [0.01, 0.05] |

**Table S4.** Summary (estimates and 95% credible intervals) of the diet model. Habitat has three levels (“exclosure, “open”, “woody”), season has two levels (“dry”, “wet”), and diet has four levels (“frugivore”, “granivore”, “invertivore”, “other”).

| **Predictor** | **mean** | **95% CrI** |
|---|---|---|
| (Intercept) | -1.78 | [-2.07, -2.44] |
| habitatwoody | 0.04 | [-0.35, 0.42] |
| habitatexclos | 0.23 | [-0.17, 0.83] |
| seasonwet | -0.45 | [-0.86, 0.24] |
| dietGranivore | -0.39 | [-0.76, 0.08] |
| dietInvertivore | -0.98 | [-1.33, 0.1] |
| dietOther | -0.99 | [-1.4, 2.26] |
| habitatwoody:seasonwet | -0.02 | [-0.62, 1.72] |
| habitatexclos:seasonwet | 0.04 | [-0.55, 1.62] |
| habitatwoody:dietGranivore | 0.17 | [-0.39, 1.32] |
| habitatexclos:dietGranivore | 0.28 | [-0.25, 3.08] |
| habitatwoody:dietInvertivore | 0.28 | [-0.23, 1.65] |
| habitatexclos:dietInvertivore | 0.36 | [-0.13, 1.21] |
| habitatwoody:dietOther | 0.21 | [-0.38, 1.3] |
| habitatexclos:dietOther | 0.55 | [-0.02, 0.65] |
| seasonwet:dietGranivore | 1.04 | [0.51, 1.47] |
| seasonwet:dietInvertivore | 0.35 | [-0.18, 1.15] |
| seasonwet:dietOther | 0.44 | [-0.15, 1.07] |
| habitatwoody:seasonwet:dietGranivore | -0.21 | [-0.97, 2.09] |
| habitatexclos:seasonwet:dietGranivore | -0.12 | [-0.88, 0.95] |
| habitatwoody:seasonwet:dietInvertivore | -0.27 | [-1.01, 1.25] |
| habitatexclos:seasonwet:dietInvertivore | -0.18 | [-0.9, 2.52] |
| habitatwoody:seasonwet:dietOther | -0.27 | [-1.1, 0.91] |
| habitatexclos:seasonwet:dietOther | -0.35 | [-1.15, 1.03] |
| Sigma[ol:(Intercept)] | 2.45 | [2.3, 1.3] |
| Sigma[landscape_id:(Intercept)] | 0.01 | [0, 0.74] |

**Table S5.** Summary (estimates and 95% credible intervals) of the migration model. Habitat has three levels ("exclosure, "open", "woody"), season has two levels ("dry", "wet"), and migration has two levels ("resident", "migrant").

| **Predictor** | **mean** | **95% CrI** |
|---|---|---|
| (Intercept) | -2.38 | [-2.52, -2.44] |
| habitatwoody | 0.2 | [0.02, 0.42] |
| habitatexclos | 0.52 | [0.34, 0.83] |
| seasonwet | 0.09 | [-0.09, 0.24] |
| migrationmigrant | -2.62 | [-3.75, 0.08] |
| habitatwoody:seasonwet | -0.24 | [-0.5, 0.1] |
| habitatexclos:seasonwet | -0.17 | [-0.43, 2.26] |
| habitatwoody:migrationmigrant | 1.44 | [0.12, 1.72] |
| habitatexclos:migrationmigrant | 1.21 | [-0.12, 1.62] |
| seasonwet:migrationmigrant | -0.28 | [-1.63, 1.32] |
| habitatwoody:seasonwet:migrationmigrant | 0.12 | [-1.62, 3.08] |
| habitatexclos:seasonwet:migrationmigrant | 0.86 | [-0.83, 1.65] |
| Sigma[ol:(Intercept)] | 2.6 | [2.44, 0.13] |
| Sigma[landscape_id:(Intercept)] | 0.01 | [0, 0.49] |

**Table S6.** Summary (estimates and 95% credible intervals) of the habitat model. Habitat has three levels ("exclosure, "open", "woody"), season has two levels ("dry", "wet"), and habitat preferences has three levels ("open", "woodland", "intermediate").

| **Predictor** | **mean** | **95% CrI** |
|---|---|---|
| (Intercept) | -2.65 | [-3.2, -2.44] |
| habitatwoody | -1.39 | [-2.25, 0.42] |
| habitatexclos | -1.81 | [-2.73, 0.83] |
| seasonwet | 0.04 | [-0.67, 0.24] |
| habitat_prefintermediate | 0.37 | [-0.22, 0.08] |
| habitat_prefwoodland | 0.2 | [-0.37, 0.1] |
| habitatwoody:seasonwet | -0.02 | [-1.13, 2.26] |
| habitatexclos:seasonwet | 0.09 | [-1.1, 1.72] |
| habitatwoody:habitat_prefintermediate | 1.65 | [0.74, 1.62] |
| habitatexclos:habitat_prefintermediate | 2.34 | [1.41, 1.32] |
| habitatwoody:habitat_prefwoodland | 1.67 | [0.78, 3.08] |
| habitatenxlos:habitat_prefwoodland | 2.46 | [1.53, 1.65] |
| seasonwet:habitat_prefintermediate | 0.3 | [-0.48, 1.21] |
| seasonwet:habitat_prefwoodland | -0.14 | [-0.88, 1.3] |
| habitatwoody:seasonwet:habitat_prefintermediate | -0.38 | [-1.61, 0.65] |
| habitatexclos:seasonwet:habitat_prefintermediate | -0.42 | [-1.7, 1.47] |
| habitatwoody:seasonwet:habitat_prefwoodland | -0.07 | [-1.26, 1.15] |
| habitatexclos:seasonwet:habitat_prefwoodland | -0.13 | [-1.35, 1.07] |
| Sigma[ol:(Intercept)] | 2.51 | [2.35, 0.65] |
| Sigma[landscape_id:(Intercept)] | 0.01 | [0, 1.15] |

**Figure S1.** Effect (estimates and credible intervals) of predictors on bird richness.

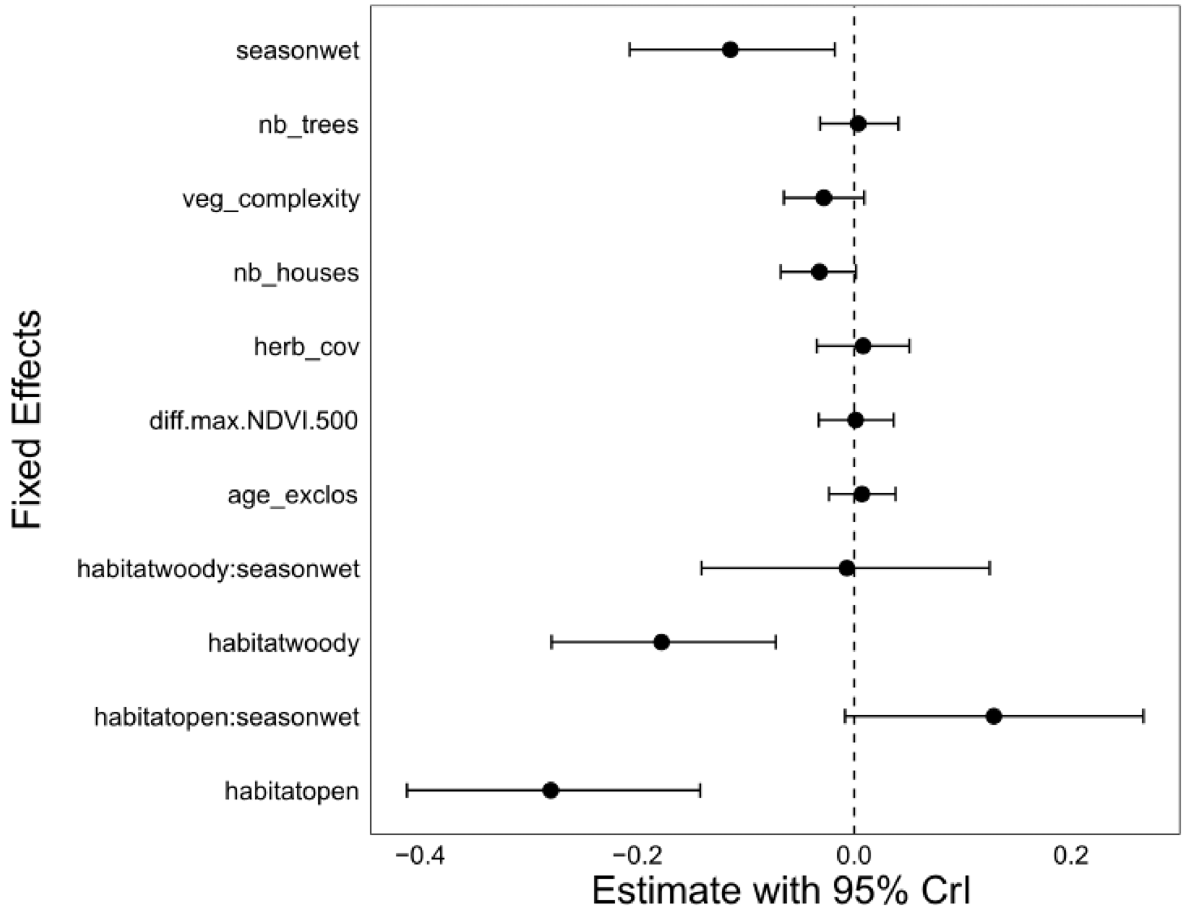

**Figure S2.** Herbaceous vegetation coverage (A) and height (B) per habitat and season. The herbaceous vegetation coverage and height were estimated in the field for each site and season within a radius of 10 meters from the center of each site. Boxplots show the median and interquartile range (box), the smallest and largest values no further than 1.5 the interquartile range (whiskers) and more extreme values (dots) of the raw data.

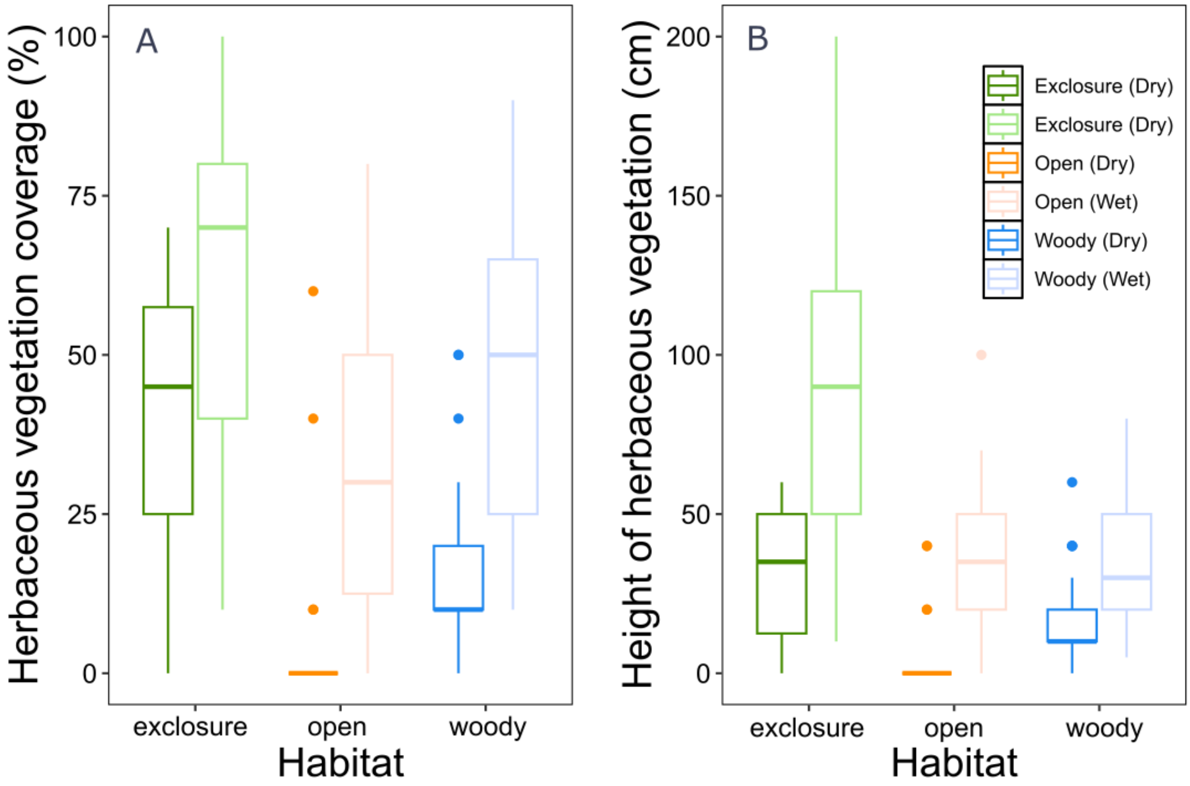

**Figure S3.** Max NDVI per habitat and season measured within 50-meters radius from the center of each site (A) and within a 500-meters radius from the center of the grazing exclusion (B). Boxplots show the median and interquartile range (box), the smallest and largest values no further than 1.5 the interquartile range (whiskers) and more extreme values (dots) of the raw data.

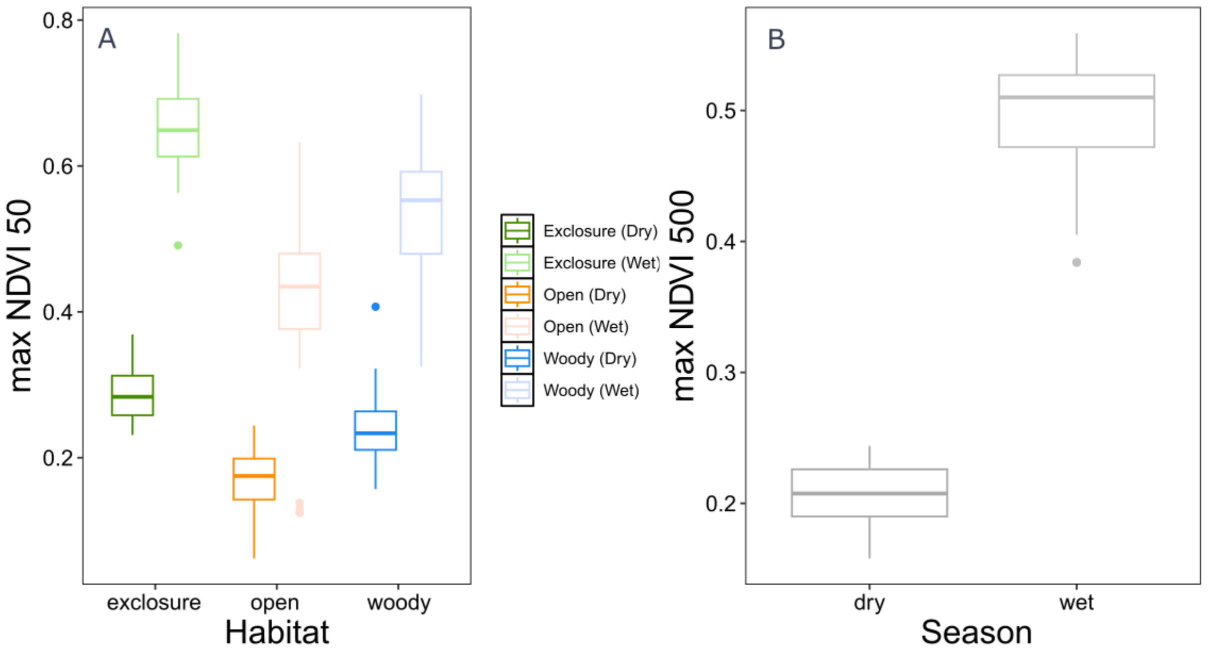

**Figure S4.** Tree richness (A), number of trees (B) and vertical vegetation heterogeneity (Shannon Index, C) in each habitat. Trees were inventoried in the field within a 30-meters radius from the center of each site. Boxplots show the median and interquartile range (box), the smallest and largest values no further than 1.5 the interquartile range (whiskers) and more extreme values (dots) of the raw data.

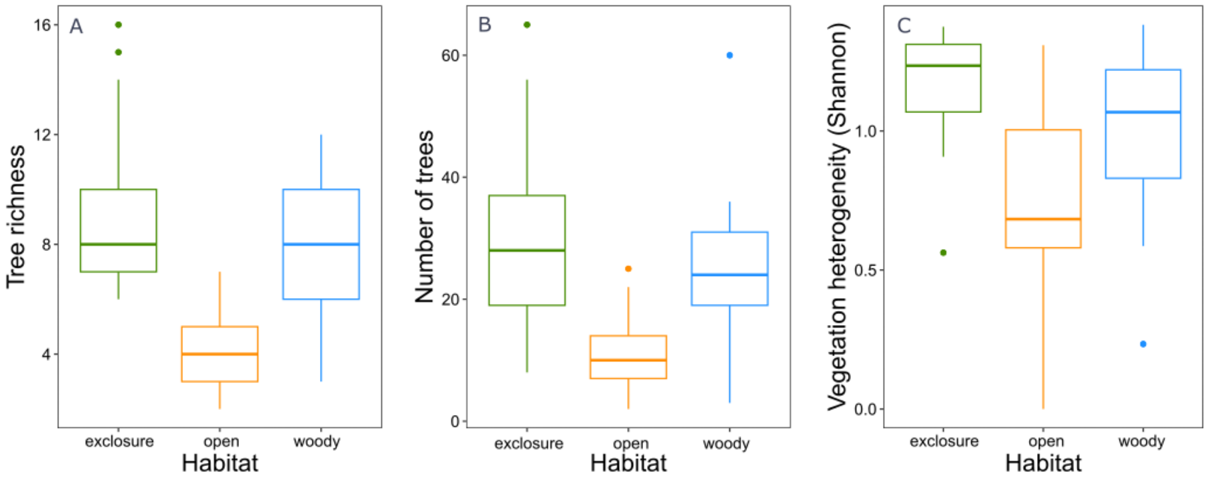

Supplement: Supplementary file 1 — Appendix S1. [file EAP-35-e70160-s001.pdf]
